# Persistent Infiltration and Impaired Response of Peripherally-Derived Monocytes after Traumatic Brain Injury in the Aged Brain

**DOI:** 10.3390/ijms19061616

**Published:** 2018-05-30

**Authors:** Austin Chou, Karen Krukowski, Josh M. Morganti, Lara-Kirstie Riparip, Susanna Rosi

**Affiliations:** 1 Brain and Spinal Injury Center, University of California, San Francisco, CA 94143, USA; austin.chou@ucsf.edu (A.C.); karen.krukowski@ucsf.edu (K.K.); josh.morganti@uky.edu (J.M.M.); lriparip@gmail.com (L.-K.R.); 2 Department of Physical Therapy Rehabilitation Science, University of California, San Francisco, CA 94143, USA; 3 Department of Neurological Surgery, University of California, San Francisco, CA 94143, USA; 4 Weill Institute for Neuroscience, University of California, San Francisco, CA 94158, USA; 5 Kavli Institute of Fundamental Neuroscience, University of California, San Francisco, CA 94158, USA

**Keywords:** traumatic brain injury, monocyte, inflammation, ageing, cognition

## Abstract

Traumatic brain injury (TBI) is a leading cause for neurological disabilities world-wide. TBI occurs most frequently among the elderly population, and elderly TBI survivors suffer from reduced recovery and poorer quality of life. The effect of age on the pathophysiology of TBI is still poorly understood. We previously established that peripherally-derived monocytes (CCR2^+^) infiltrate the injured brain and contribute to chronic TBI-induced cognitive deficits in young animals. Furthermore, age was shown to amplify monocyte infiltration acutely after injury. In the current study, we investigated the impact of age on the subchronic response of peripherally-derived monocytes (CD45^hi^; CCR2^+^) and their role in the development of chronic cognitive deficits. In the aged brain, there was a significant increase in the number of peripherally-derived monocytes after injury compared to young, injured animals. The infiltration rate of peripherally-derived monocytes remained elevated subchronically and corresponded with enhanced expression of CCR2 chemotactic ligands. Interestingly, the myeloid cell populations observed in injured aged brains had impaired anti-inflammatory responses compared to those in young animals. Additionally, in the aged animals, there was an expansion of the blood CCR2^+^ monocyte population after injury that was not present in the young animals. Importantly, knocking out CCR2 to inhibit infiltration of peripherally-derived monocytes prevented chronic TBI-induced spatial memory deficits in the aged mice. Altogether, these results demonstrate the critical effects of age on the peripherally-derived monocyte response during the progression of TBI pathophysiology.

## 1. Introduction

Traumatic brain injury (TBI) is a leading cause of neurological disability and a major risk factor for the development of neurodegenerative diseases and dementia [1,2]. The higher incidence of TBIs among the elderly is a rapidly emerging health issue as the world population grows and the average human lifespan lengthens [3]. Rates of TBI-related mortality and hospitalization are higher for elderly patients, and this population also suffers from worse cognitive recovery and poorer quality of life post-injury [4,5,6]. This disparity between elderly and younger populations is significant even after mild TBIs [7]. Beyond recognizing age as a prognostic factor for TBI outcomes [8], research to identify TBI mechanisms affected by age is imperative as the number of elderly TBI patients inevitably increases.

A few studies have established the efficacy of rodent TBI models for investigating the underlying effects of age on TBI outcomes [9,10]. It has been shown that TBIs result in greater neurodegeneration and reduced behavioral recovery in aged mice compared to young mice [10,11]. Age-associated increases of injury cavitation size and neuronal death post-TBI have been shown to correlate with an amplified acute inflammatory cytokine response at 2 days post-injury (2 dpi) [11,12,13]. There is also a corresponding increase in microglia/macrophage activation that persists several weeks post-injury as exhibited by upregulated activation markers and amoeboid morphology [14,15,16].

Infiltrated macrophages—peripherally-derived monocytes that have entered the injured brain and differentiated to activated macrophages—play a significant role in TBI-induced inflammation and cognitive deficits in adult animals [17,18,19]. Peripherally-derived monocytes are defined by their CCR2 surface expression; the CCR2 receptor and its primary ligand, CCL2, represent the main signaling pathway of peripherally-derived monocyte infiltration (referred to as “monocyte infiltration”) [20]. After TBI, CCL2 is upregulated in both human patients and rodent models of injury [19,21,22]. Knocking out or pharmacologically inhibiting CCR2 prevents 80–90% of monocyte infiltration, reduces inflammatory cytokine expression, preserves neuronal density, and reduces the development of chronic learning impairments in young animals after TBI [17,18,23]. Our lab has shown that in aged brains, peripherally-derived monocyte recruitment within 24 h of injury is seven times greater compared to young animals [15]; accordingly, we hypothesized that the peripherally-derived monocyte population in the brain contributes to the age-driven differences after injury.

In this study, we demonstrated that age increases the peripherally-derived monocyte population in the injured brain for up to a week post-injury. Furthermore, the amplified rate of infiltration in aged mice persists at 4 days post injury, and age increases the subchronic expression of the CCR2 ligands responsible for monocyte infiltration. Notably, there is also significant expansion of the peripherally-derived monocyte population in the blood of the aged animals after TBI. Importantly, we found that age impairs the anti-inflammatory response of myeloid cells during the resolution of injury-driven inflammation. Preventing the infiltration of peripherally-derived monocytes through a CCR2 knockout in aged mice ameliorates the development of TBI-induced spatial memory deficits which highlights the impact of the aged peripheral immune system on TBI outcomes.

## 2. Results

### 2.1. Age Increases Peripherally-Derived Monocytes in the Injured Brain at 4 and 7 Days Post-Injury

**Figure 1:**
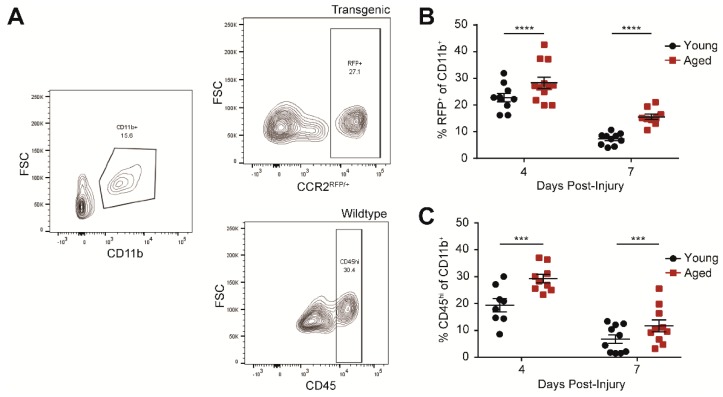
Age increases peripherally-derived monocytes (CD11b^+^CD45^hi^ or CD11b^+^CCR2^+^) at 4 and 7 days post-traumatic brain injury (TBI). (**A**) Representative flow cytometry profiles for the peripherally-derived monocyte population in the brain. CD11b^+^ is gated from the live, single cell population (left). Peripherally-derived monocytes are gated as either RFP^+^ (top right) or CD45^hi^ (bottom right) from CCR2^rfp/+^ transgenic or wildtype animals respectively. Example images are from aged animals at 4 days post-injury (dpi). (**B**) Percent CCR2^rfp/+^ of the CD11b^+^ population at 4 and 7 dpi. Age increases CCR2^+^ cells at 4 and 7 dpi after TBI. Data are means ± SEM (*n* = 9–12; two-way ANOVA, main effects of age and time, **** *p* < 0.0001). (**C**) Percent CD45^hi^ of the CD11b^+^ population at 4 and 7 dpi. Age increased CD45^hi^ cells at both time points after TBI. Data are means ± SEM (*n* = 8–10; two-way ANOVA, significant main effects of age and time, no significant interaction, *** *p* < 0.001).

We have previously shown that there is exacerbated infiltration of peripherally-derived monocytes—CD11b^+^, F4/80^hi^, CCR2^+^—in the aged brain acutely after injury [15]. To determine whether the effect of age persists subchronically, we quantified peripherally-derived monocytes in the brain at 4 and 7 days post-injury (dpi) in young and aged animals. As in our previous studies, we utilized an established transgenic line to label the CCR2^+^ monocytes with red fluorescent protein (RFP) in the injured brains (Figure 1A, top) [18]. We measured an increase in the number of peripherally-derived monocytes at 4 and 7 dpi compared to the younger counterparts (Figure 1B). Because of the limited number of aged transgenic animals available, we also verified our results in wildtype animals by gating for CD45^hi^ monocytes (Figure 1A, bottom); CD45^hi^ and CCR2 identify the same monocyte population in the injured brain (Figure S1A,B). Indeed, we confirmed the increase of peripherally-derived monocytes in aged wildtype animals after injury compared to young wildtype animals (Figure 1C).

### 2.2. Age Exacerbates Subchronic Infiltration of Peripherally-Derived Monocytes to the Injured Brain

**Figure 2:**
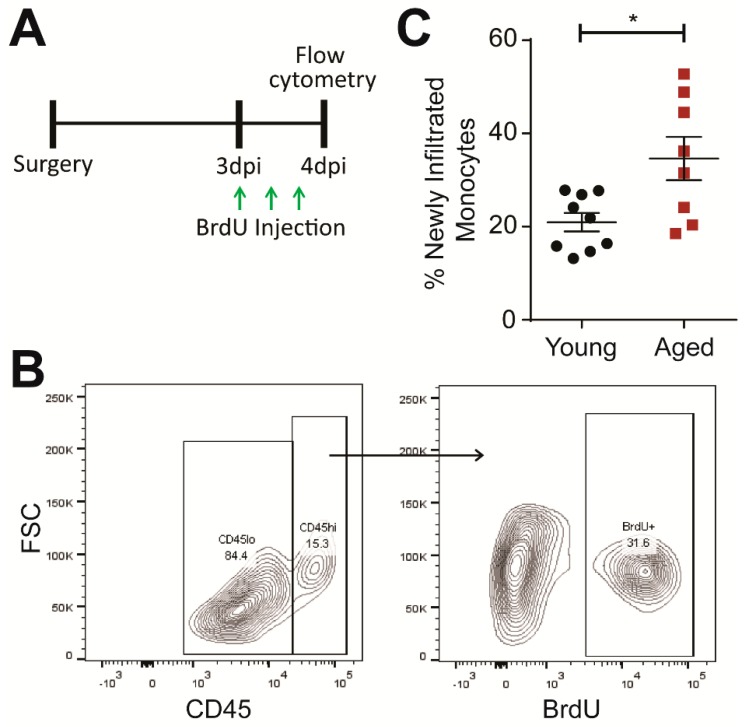
Age increases infiltration of peripherally-derived monocytes (CD11b^+^, F4/80^+^, CD45^hi^) into the brain at 4 dpi. (**A**) Experiment design of BrdU injections. Animals were given three injections of 100 mg/kg BrdU every 8 h starting at 3 dpi. The animals were then euthanized, and the brains were collected for flow cytometry at 4 dpi. (**B**) Representative flow cytometry profiles for recently-infiltrated peripherally-derived monocytes in the TBI brain. CD45^hi^ monocytes were first gated from the CD11b^+^, F4/80^+^ population (left). The BrdU signal was then gated from the peripherally-derived monocyte population. Example images are from an aged animal. (**C**) Percentage of infiltrated monocytes (CD45^hi^) with BrdU^+^ signal. Age significantly increased the number of newly infiltrated monocytes between 3 and 4 dpi. Data are means ± SEM (*n* = 8–9; Student’s *t*-test, * *p* < 0.05).

Next, we investigated whether the age-driven increase of monocyte infiltration into the injured brain is limited to the first 24 h or persists subchronically. Using BrdU, an analog of thymidine that is incorporated into proliferating cells during DNA replication, we labeled new peripherally-derived monocytes between 3–4 dpi (Figure 2A,B). Peripherally-derived monocytes originate from the bone marrow [22,24], and thus, any observed BrdU-labeled monocytes in the injured brain will proliferate in the bone marrow, enter the circulation, and then infiltrate the injured brain after 3 dpi. This established monocyte development pathway allows us to measure monocyte infiltration dynamics between 3–4 dpi.

With this approach, we detected a higher percentage of BrdU-labeled monocytes in the aged brain at 4 dpi (Figure 2C). This result indicates that age indeed exacerbates monocyte infiltration subchronically. Furthermore, we measured an increase in proliferating microglia—the resident macrophage population in the brain defined as CD11b^+^ CD45^lo^—in aged animals corresponding with previous work showing that age enhances the microglial response (Figure S2A,B) [14,16].

### 2.3. Age Increases CCR2 Signaling in the Injured Brain

**Figure 3:**
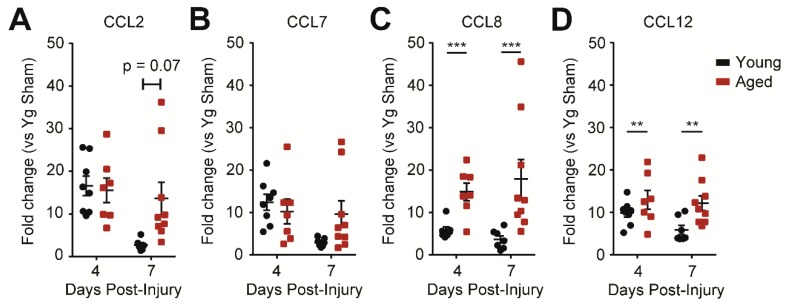
Aged animals have greater CCR2 ligand expression at 4 and 7 days post-injury (dpi). (**A**,**B**) Quantitative PCR data for CCL2 and CCL7 expression in the hippocampus ipsilateral to injury. At 4 and 7 dpi, there was no difference between aged and young animals in CCL2 and CCL7 expression at 4 dpi, but there was a trend for decreased expression in young animals by 7 dpi. (**C**,**D**) Quantitative PCR data for CCL8 and CCL12 expression in the hippocampus ipsilateral to injury. Aged animals have significantly higher CCL8 and CCL12 expression at both 4 and 7 dpi (*n* = 7–9; two-way ANOVA, significant main effect of age only, no significant interactions, ** *p* < 0.01, *** *p* < 0.001).

To investigate possible mechanisms underlying the difference in monocyte infiltration between young and aged mice, we measured the expression of the CCR2 ligand family—CCL2, CCL7, CCL8, and CCL12—by qPCR. We found that CCL8 and CCL12 levels were higher in the aged animals at both 4 and 7 dpi compared to young injured animals at the respective time points. For CCL2 and CCL7 there was a trend for aged animals to maintain upregulation while young animals began to downregulate the two chemokines by 7 dpi (Figure 3A–D). The increases in the chemotactic ligand expressions may contribute to the exacerbated infiltration of CCR2^+^ peripherally-derived monocytes in the aged animals.

### 2.4. Age Increases CCR2^+^ Monocytes in the Blood Population after Injury

**Figure 4:**
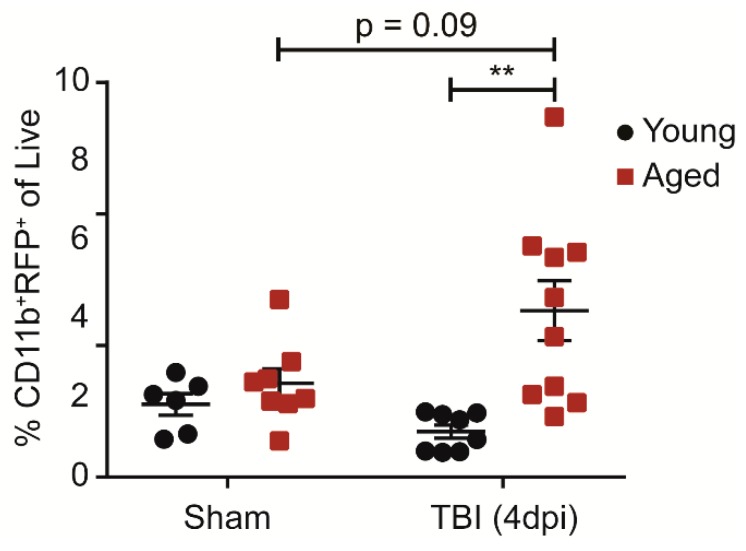
Age increases CCR2^+^ monocytes in the blood after TBI. Percentage of CD11b^+^, CCR2^+^ monocytes of all single, live cells in 100 μL of blood. Age increases the CCR2^+^ monocyte population at 4 dpi. (*n* = 6–10; two-way ANOVA, significant main effect of age with significant interaction; Bonferroni’s post hoc test, ** *p* < 0.01).

We next determined whether age-induced differences in the CCR2^+^ monocyte response after injury is restricted to the brain or if it is reflected globally in the blood and the bone marrow. Aged animals had an increase in CCR2^+^ monocytes in the blood 4 days after injury whereas the young animals did not (Figure 4). To further investigate whether peripheral monocyte expansion is the result of increased proliferation in the bone marrow, we used EdU, an alternative to BrdU, to label new monocytes between 3–4 dpi. We did not observe an age difference in newly-proliferated monocytes in the bone marrow (Figure S3A). To then test if age affects the rate of monocyte egression from the bone marrow, we examined EdU-labeled monocytes in the blood and once again did not observe significant differences (Figure S3B). These results suggest that age drives the expansion of peripheral monocytes after injury that either occurs gradually or before 3 dpi.

### 2.5. Age Modifies the Subchronic Anti-Inflammatory Response to TBI

**Figure 5:**
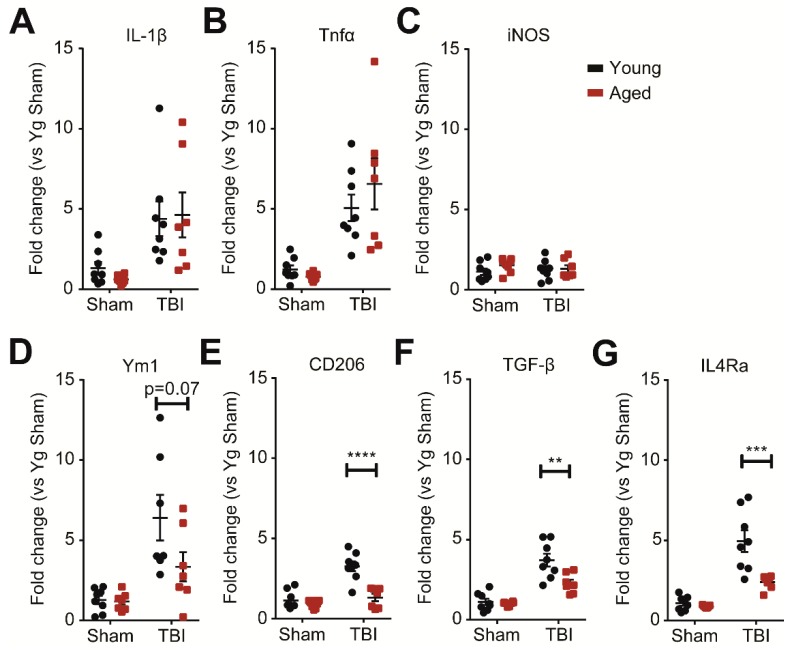
Age decreases the subchronic anti-inflammatory responses of monocytes and microglia (myeloid cells) at 7 dpi. (**A**–**C**) Quantitative PCR data for IL-1β, Tnf-α, and iNOS as markers representative of pro-inflammation. Myeloid cells were isolated from whole brain by percoll gradient and processed for qPCR. TBI increases the expression of IL-1β and Tnf-α at 7 dpi with no difference due to age (*n* = 7–8; two-way ANOVA; IL-1β, Tnf-α: main effect of TBI only). (**D**–**G**) Quantitative PCR data for Ym1, CD206, TGF-β, and IL4Ra as representative markers of anti-inflammation. TBI increases the expression of the markers at 7 dpi with significantly less expression in the aged animals. (*n* = 7–8; two-way ANOVA; Ym1: significant main effect of TBI with trends for significant effects of age and interaction; CD206, TGF-β, IL4Ra: significant main effects of TBI and age with significant interaction; Bonferroni’s post hoc test, ** *p* < 0.01, *** *p* < 0.001, **** *p* < 0.0001).

In addition to affecting the infiltration of monocytes, age acutely enhances inflammatory cytokine expression within the first 24 h after injury [14,15]. As we observed age-driven increases in both peripherally-derived monocyte and resident microglia populations after injury, we further ascertained whether age would affect the inflammatory profiles of monocytes and microglia (myeloid cells) at 7 dpi. We isolated the myeloid cells from the injured brain by percoll gradient and quantified inflammatory marker expression by qPCR. We found that the pro-inflammatory markers, IL-β, Tnf-α, and iNOS, were similarly upregulated between aged and young animals after TBI (Figure 5A–C). However, the expression of the anti-inflammatory markers, Ym1, CD206, TGF-β, and IL4Ra, was significantly reduced in the aged animals (Figure 5D–G). Thus, age selectively impairs the anti-inflammatory response that is necessary for resolving immune activation after injury without affecting TBI-induced pro-inflammatory markers.

### 2.6. CCR2 Knockout Prevents TBI-Induced Memory Deficits in Aged Animals

We previously showed that inhibiting the infiltration of peripherally-derived monocytes via CCR2 signaling prevented the development of chronic, TBI-induced cognitive deficits in young mice [18]. Additionally, blocking monocyte infiltration with a CCR2 antagonist successfully reduced age-related exacerbation of inflammation at 24 h post-injury. These results support the notion that the targeting of CCR2 infiltration can be used to ameliorate TBI-induced cognitive deficits in aged mice [15]. In the present study, we utilized the radial arm water maze (RAWM)—a hippocampal-dependent spatial learning and memory assay—at 30 dpi to determine the effect of CCR2 knockout on TBI-induced cognitive outcomes in aged animals.

**Figure 6:**
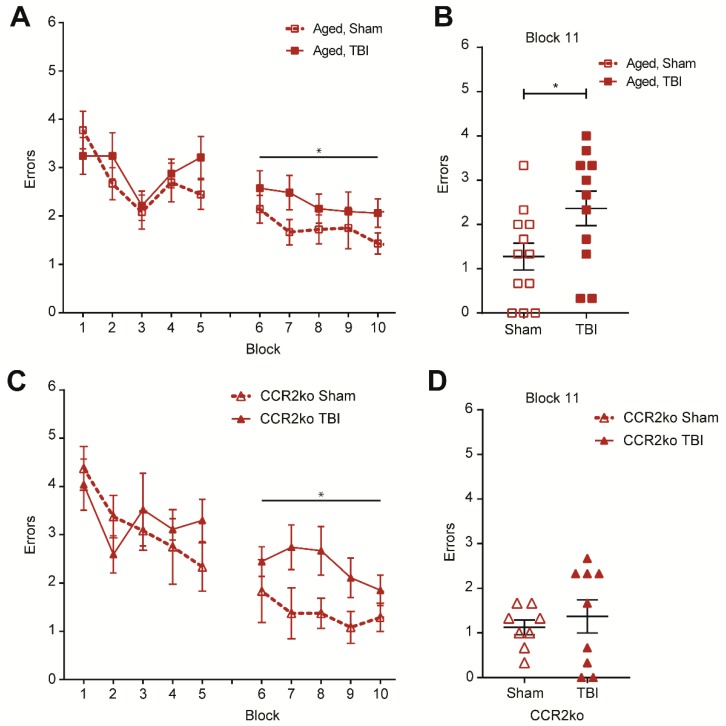
Age exacerbates TBI-induced spatial learning and memory deficits on the radial arm water maze (RAWM) at 30 dpi. Knockout of CCR2 partially prevents spatial learning deficits. (**A**) Animals were trained on 15 trials per day (every three trials were averaged to calculate a block for a total of five blocks each training day) of RAWM starting at 28 dpi through to 29 dpi. TBI significantly increased errors in aged animals on blocks 6–10 and on memory block 11 (*n* = 11–12; two-way ANOVA, main effects of TBI only, * *p* < 0.05). (**B**) At 30 dpi (block 11), animals were tested for their memory of the escape platform location. TBI significantly increased the number of errors committed by aged animals during the memory test (Student’s *t*-test, * *p* < 0.05). (**C**) CCR2 knockout animals with either sham or TBI surgery were similarly tested on the RAWM at 28–30 dpi. TBI animals committed more errors during learning (blocks 6–10) than the sham controls (*n* = 8–9; two-way ANOVA, main effects of TBI only, * *p* < 0.05). (**D**) Individual animal performance during the memory test (block 11; 30 dpi) of CCR2 knockout animals with either sham or TBI surgery. No significant differences were observed.

During the RAWM assay, animals learn to locate a hidden platform in one of eight arms using visual cues in the room. The number of incorrect entries prior to successfully locating the escape platform is used as a metric of spatial learning and memory. Aged, injured wildtype mice performed significantly worse, as exhibited by more errors committed during the second day of training (Figure 6A). After two training days, animals were tested on a memory test block, and animals that had received a TBI 30 days prior committed significantly more errors during the memory test (Figure 6B). In line with published studies showing that age impairs cognitive function [25,26], we found that sham and injured aged animals performed significantly worse on the RAWM compared to young animals (Figure S4). Similar to the wildtype counterparts, aged, injured CCR2 knockout mice exhibited deficits during training (Figure 6C). However, during the memory test, the CCR2 knockout TBI cohort performed at levels comparable to the sham animals, suggesting that blocking monocyte infiltration could at least partially rescue performance in the RAWM assay and prevent TBI-induced memory impairment (Figure 6D).

## 3. Discussion

The current results demonstrate that age exacerbates peripherally-derived monocyte infiltration and modifies the subchronic anti-inflammatory response resulting in worse cognitive outcomes after injury. We reported an age-driven increase in peripherally-derived monocytes both in the blood and in the injured brain with a corresponding elevation in CCR2 chemotactic ligands. Notably, blocking monocyte infiltration by CCR2 knockout was shown to prevent TBI-induced spatial memory deficits (Figure S5).

The expansion of myeloid populations in the injured brain is known to be affected by age. Specifically, there is further upregulation of the myeloid markers, CD11b and Iba1, in aged compared to young animals. The increases in both CD11b and Iba1 have been shown to persist chronically in aged animals [16]. However, the authors of this previous study did not distinguish between peripherally-derived monocytes and resident microglia. In the present study, we investigated how the kinetics of monocyte infiltration are affected by age at subchronic time points after injury. We demonstrated that there is a greater number of peripherally-derived monocytes in the aged brain at both 4 and 7 dpi. Moreover, we found that age-exacerbated monocyte infiltration persists subchronically.

Importantly, we observed that aged animals had an expansion of CCR2^+^ monocytes in the blood after injury that was absent in young, injured animals. This novel observation suggests that monocyte expansion in the blood may also serve an age-specific biomarker for TBI outcomes. Current blood biomarkers for TBI comprise only proteins that exit the brain into the blood, without obvious mechanisms on how the biomarkers directly affect the TBI outcome [27,28]. In contrast, our lab and others have demonstrated that peripherally-derived monocytes contribute to chronic TBI-induced deficits [17,18]. Interestingly, a study on human patients of varying ages found a small but significant increase in blood monocytes within the initial 24 h after injury [29,30]. Our findings suggest that their results may have been partially masked by the wide age range (24 to 62 years old) of their subjects who were not stratified by age. While our study does not have the power to conclusively distinguish between an acute burst of monocyte proliferation and egress into the blood or a more gradual process, it is possible that elderly human patients could exhibit significantly more blood monocytes at subchronic rather than acute time points. Our data also does not eliminate the possibility that decreases in other blood lymphocyte populations may contribute to our observations. Overall, more comprehensive studies to correlate the peripheral monocyte count and TBI outcomes are necessary to prove the utility of blood monocytes as a biomarker for brain injury in elderly patients.

While the primary source of CD45^hi^ and CCR2^+^ monocytes is the bone marrow [22,24], sites such as the brain that have a resident macrophage population may present a permissive environment for proliferation of peripherally-derived monocytes. In fact, peripherally-derived monocytes and resident microglia already share similar markers and functions despite having distinct origins [31,32]. After infiltration, peripherally-derived monocytes downregulate the expression of CCR2 and take on a microglia-like state, for example, expressing the microglia marker CX3CR1 [18,33]. Therefore, it is possible that already-infiltrated monocytes proliferate and give rise to some of the BrdU-labeled monocytes observed in the injured brain. The proliferation of peripherally-derived monocytes has been observed in the peritoneum—another region with resident tissue macrophages—during peritonitis [34]. Future studies should investigate whether the injured brain environment allows peripherally-derived monocytes to take on a proliferative capacity similar to microglia [35,36]. Furthermore, given that our study and others have consistently observed an age-induced increase in microglia proliferation after injury [14,16,37], the proliferation of peripherally-derived monocytes in the injured brain may also be affected by age.

We previously reported that in the aged brain, there is an increase in the CCR2 ligands, CCL7, CCL8, and CCL12, at 24 h post-injury [15]. Here, we demonstrated that the age-driven differences in CCL8 and CCL12 persist at 4 and 7 dpi. This increase potentially contributes to the exacerbated monocyte infiltration observed in the aged, injured brain. Interestingly, while young and aged animals express the same levels of CCL2 and CCL7 at 4 dpi, we observed a trend where young animals downregulate, while aged animals maintain, this expression at 7 dpi. These findings present the possibility that by 7 dpi, monocyte infiltration may taper off in young animals, but persist in aged animals. Notably, in a previous study where we gated only for the F4/80^hi^ subpopulation of peripherally-derived monocytes [18], we reported that monocyte infiltration ended by 48 h. However, when we included all F4/80^+^ cells in the current study, we detected ongoing monocyte infiltration at 4 dpi in young animals. Further studies are required to pinpoint the halt in monocyte infiltration in both age groups and to determine whether age simply amplifies infiltration or extends the window of monocyte infiltration. Validation studies utilizing immunohistochemistry could provide additional insight into whether specific regions of the aged brain are more susceptible to exacerbated monocyte infiltration. Additionally, while our gating scheme predominantly selects for the monocyte population, our data does not fully exclude other cell types. For example, neutrophils also express CD11b, CD45^hi^, and CCR2 and acutely infiltrate the injured brain in young animals [17,38]. While neutrophil infiltration dominates at 24 h and tapers off by 3–7 dpi, future studies should identify if the neutrophil response is similarly affected in aged mice. Previous studies have shown that age increases both pro- and anti-inflammation gene expressions within the first 24 h after injury [11,14,15]. At 7 dpi, our data suggest that pro-inflammatory expression is similar between young and aged animals, but anti-inflammatory gene expression is impaired in the myeloid cells of aged animals. The two profiles broadly indicate the microglia/macrophage polarization into pro- and anti-inflammatory phenotypes [39,40]. While we have previously shown that the myeloid population can express both phenotypes simultaneously after TBI [40,41,42], the decrease in the anti-inflammatory markers, Ym1 and CD206, in the aged TBI animals suggest less overall anti-inflammatory functionality [43]. Indeed, the aged brain already predisposes myeloid cells towards pro-inflammatory, and away from anti-inflammatory, gene expression [44]. Furthermore, the observed decreases in TGF-β and IL-4Ra—a cytokine and receptor that promote an anti-inflammatory phenotype—suggest general impairment of anti-inflammatory polarization rather than of specific cytokine pathways [39,45]. The age-driven bias away from the anti-inflammatory myeloid phenotype may further exacerbate chronic inflammation after injury to worsen TBI outcomes [14,18]. As a comparison, peripherally-derived monocytes after stroke or spinal cord injury exhibit anti-inflammatory and neuroprotective functions that ultimately improve long term functional recovery [46,47,48]. Though monocyte infiltration exacerbates chronic deficits after TBI [17,19,23], we previously showed that peripherally-derived monocytes specifically are associated with the spike of anti-inflammatory markers at 7 dpi in young mice [18]. This would suggest that the deficits in anti-inflammation in the aged population could be attributed to the impairment of peripherally-derived monocytes in particular. Instead of dampening pro-inflammation, treatments that restore or augment the anti-inflammatory pathways of peripherally-derived monocytes might prove more effective for treating TBIs in the elderly population. Importantly, our results also suggest that such approaches could be applicable over a wider window of time post-injury.

The prevention of CCR2^+^ monocyte infiltration in young animals significantly reduces both pro- and anti-inflammatory markers and ultimately improves cognitive outcomes after TBI [17,18]. Blocking monocyte infiltration with a CCR2 antagonist likewise reduces acute inflammation in aged, injured animals [15]. In our behavioral experiment, we first found that age significantly impaired the RAWM performance of both sham and injured animals, which agrees with published work where age alone has been shown to worsen cognition [25,26]. The prevention of monocyte infiltration by knocking out CCR2 ameliorated TBI-induced deficits on spatial memory in aged, injured animals. However, we still observed learning impairments during the RAWM training days (blocks 1–10). Importantly, this approach only accounted for the involvement of peripherally-derived monocytes and largely ignored microglia. Injury-induced microglia activation also contributes to chronic inflammation and cognitive deficits, similar to peripherally-derived monocytes [37,38]. How age affects the microglia response after injury has not been distinguished from the peripherally-derived monocyte response, and the brain resident population may be able to compensate for the lack of monocyte infiltration shown in our study. Additionally, other secondary injury mechanisms could have worsened TBI outcomes in parallel to monocyte infiltration in the aged mice which would not have been rescued by our approach.

In conclusion, our results significantly extend our knowledge on how age affects TBI inflammation and peripherally-derived monocyte response and provides new avenues for improving treatment for elderly TBI patients. Namely, we highlighted the possibility of peripheral monocytes as an age-specific biomarker and emphasized the impairment of anti-inflammatory pathways after TBI by age. We are hopeful these findings may accelerate translational research to improve patient outcomes for the population most at risk for TBIs.

## 4. Materials and Methods

### 4.1. Animals

All experiments were conducted in accordance with the National Institutes of Health *Guide for the Care and Use of Laboratory Animals* and were approved by the Institutional Animal Care and Use Committee of University of California San Francisco (Protocol number: 170302, Approved: July 2017–2020; Protocol number: 106982, Approved: June 2014–2017). Adult males, aged 3–6 months (young) and 20–25 months (C57B6/J) were purchased from Jackson Laboratory (Bar Harbor, ME) and the National Institute on Aging Animal Colony, respectively. Young and aged *CX3CR1^GFP/+^CCR2^RFP/+^* (double heterozygous (Dbl-Het)), *CCR2^RFP/+^,* and *CCR2^RFP/RFP^* (effectively CCR2^−/−^) mice were bred and aged as previously described [33] and genotyped using a commercial service (Transnetyx). Mice were group housed in environmentally-controlled conditions with a reverse light cycle (12:12 h light:dark cycle at 21 ± 1 °C) and provided food and water ad libitum. The experimental time points are presented in Supplementary Figure S5.

### 4.2. TBI Surgical Procedure

Animals were anesthetized and maintained at 2% isoflurane and secured to a stereotaxic frame with non-traumatic ear bars. The hair on the scalp was removed by shaving with an electric razor. Eye lubricant was applied to their eyes, and betadine and 70% alcohol were applied to the scalp. A midline incision was made to expose the skull.

A unilateral TBI was induced via the controlled cortical impact (CCI) model in the parietal lobe, as previously described [15,49]. Mice received a craniectomy ~3.5 mm in diameter using an electric microdrill at the following coordinates: −2.00 mm anteroposterior and +2.00 mm mediolateral with respect to bregma. Any animal with excessive bleeding due to disruption of the dura was removed from the study. After the craniectomy, a contusion was delivered using a 3 mm convex tip attached to an electromagnetic impactor (Impact One, Leica). The impact parameters were set to a contusion depth of 0.95 mm (from dura) at a velocity of 4.0 m/s and sustained for 300 ms. After the CCI, the scalp was either sutured for survival after a week or closed with wound clips for experiments at seven days post-injury (dpi) or shorter. Sham animals underwent a similar procedure but with only slight drilling without removal of the skull.

Post-surgery, animals were placed in a cage on top of a heatpad until they were fully ambulatory and recovered. After recovery, animals were returned to their home cage and monitored for normal behavior and weight maintenance throughout the duration of the experiments.

### 4.3. BrdU/EdU Injection

A 10 mg/mL solution of BrdU (Sigma-Aldrich, St. Louis, MO, USA) or molar equivalent of EdU (ThermoFisher, Waltham, MA, USA) was prepared fresh in sterile PBS (Gibco, Grand Island, NY, USA) for each injection time point. Animals were injected intraperitoneally at 100 mg BrdU/kg (or molar equivalent for EdU) per injection. For Figure 2, animals were injected with BrdU starting at 3 dpi every 8 h and sacrificed 24 h after the first injection (4 dpi). Animals in the experiments shown in Figure 4 were given EdU injections every 12 h starting at 3 dpi for a total of two injections.

### 4.4. Tissue Collection

All mice were euthanized with a mixture of ketamine (80–100 mg/kg)/xylazime (5–10 mg/kg) in accordance with standard animal protocols. For the flow cytometry endpoints, blood was drawn via cardiac puncture and mixed with EDTA to prevent coagulation (1.5 μL 0.5M EDTA per 100 μL blood). Animals were then perfused transcardially with cold Hank’s balanced salt solution without calcium and magnesium (HBSS; Gibco, Grand Island, NY, USA). Immediately after perfusion, mice were decapitated, and their brains were extracted into HBSS and kept on ice for flow cytometry. For qRT-PCR endpoints, mice were perfused with phosphate buffered saline without calcium chloride and magnesium chloride (PBS; Gibco, Grand Island, NY, USA) and the hippocampi were dissected out and snap-frozen on dry ice.

### 4.5. Flow Cytometry

The olfactory bulbs and cerebellums were removed prior to processing brain tissue. The remaining tissues were diced in plastic weigh boats with a razor blade and mechanically homogenized with glass dounce tissue grinders (Kimble Kontes, Rockwood, TN, USA). The cell suspension was then filtered through 70 μm cell strainers (Fisherbrand, Pittsburgh, PA, USA). For the BrdU study, myelin was removed by adding percoll (Sigma-Aldrich, St. Louis, MO, USA) to create a 30% percoll solution. This was centrifuged at 500× *g* for 30 min to remove the supernatant. For all other flow experiments with brain samples, myeloid isolation was performed by layering a 70% percoll solution under the 30% percoll layer to form a gradient. After centrifugation, 3 mL–which contained the myeloid populations—was collected at the interface between the two densities [15]. Samples were then washed with HBSS before proceeding to staining.

Blood samples were processed by adding 1.5 mL of BD Pharm Lyse lysing buffer (BD Biosciences, San Jose, CA, USA) and incubating on ice for one minute before centrifugation. The lysis step was repeated with another 0.5 mL of lysing buffer to remove most of the red blood cells.

All washes and antibody dilutions were done in staining buffer made of 2% fetal bovine serum (FBS; Gibco, Grand Island, NY, USA) in PBS. Fc receptor blocking was performed before all staining procedures using an anti-CD16/32 antibody (1:100; BD Pharmingen, Franklin Lakes, NJ, USA). The following reagents were used for labeling the monocytic populations across all experiments: CD11b Alexafluor 700 (1:1000; BD Pharmingen, Franklin Lakes, NJ, USA) and F4/80 APC (1:200; Invitrogen, Waltham, MA, USA). CD45 FITC (1:100; BD Pharmingen, Franklin Lakes, NJ, USA) and CD45 Brilliant Violet 711 (1:100; BD Horizon, Franklin Lakes, NJ, USA) were used to differentiate resident microglia and peripherally-derived monocytes in non-BrdU and BrdU experiments respectively. ZombieViolet (1:100; BD Biolegend, Franklin Lakes, NJ, USA) or 7AAD (Sigma-Aldrich, St. Louis, MO, USA) was used to label live/dead populations. BrdU staining was performed utilizing a FITC BrdU Flow Kit (BD Pharmingen, Franklin Lakes, NJ, USA). EdU staining was performed with a ClickIt Plus Pacific Blue Flow Cytometry Kit (Invitrogen, Waltham, MA, USA). Spectral compensation was achieved using polystyrene microparticles (BD Pharmingen, Franklin Lakes, NJ, USA) in combination with the listed antibodies. Analysis was done on a FACS Aria III cell sorter (BD Biosciences, San Jose, CA, USA). Flow cytometric data was analyzed using FlowJo (v10.3, Treestar, Ashland, OR, USA).

### 4.6. qRT-PCR

Gene expression analyses were performed on dissected ipsilateral hippocampi (for CCR2 ligands) or isolated myeloid cells from the injury hemisphere (for inflammatory markers). Myeloid cell isolation was performed by mechanically homogenizing the tissue with glass dounce tissue grinders (Kimble Kontes, Rockwood, TN, USA), filtering through 70 μm cell strainers (Fisherbrand, Pittsburgh, PA, USA) and separating the population in a percoll gradient, as described above [15]. RNA isolation and cDNA conversion were performed, as previously described, with Qiazol reagent and RNEasy mini-columns (QIAGEN, Valencia, CA, USA) [18]. RNA concentration was determined via NanoDrop (Thermo Scientific, Waltham, MA, USA). For each sample, 300 ng of total RNA was reverse transcribed using the High-Capacity cDNA Reverse Transcription Kit (Applied Biosystems, Foster City, CA, USA). The genes of interest were analyzed using SYBR Green Master Mix (Applied Biosystems, Foster City, CA, USA) on a Stratagene Mx3005P Real-Time PCR system. The relative target gene expression was determined by the 2^−ΔΔ*C*t^ method normalized to beta-actin expression. The primer sequences used were as previously described [15].

### 4.7. Radial Arm Water Maze

At 28 dpi, animals were tested on the radial arm water maze (RAWM), as previously described, to test spatial learning and memory [50]. The maze consisted of a circular pool with a diameter of 118.5 cm with eight arms of 41 cm each in length. The escape platform could be moved to the end of any of the arms. The pool was filled with water, and paint (Crayola, 54-2128-053) was added to make the water opaque. Visual cues were placed around the room such that they were visible to animals in the maze. Animals ran 15 trials during two days of training (blocks 1–10) and then 3 trials during the memory test (block 11). On the first day of training, the escape platform was made visible every other trial by placing a flag on the platform. The escape platform alternated between visible and hidden for the first 12 trials and was hidden for trials 13–15. For the rest of the assay, the escape platform remained hidden.

During a trial, animals were placed in a pseudo-random arm not containing the escape platform. Animals were given one minute to explore the maze and locate the escape platform. If they successfully swam to the platform, they were returned to their holding cage after a 10 s interval. For failed trials, animals were guided to the escape platform and then returned to the holding cage 10 s later. The escape platform location was the same for each individual animal. The start arm was randomized such that each non-escape arm was used before any repetition. The escape platform location was randomly assigned for each animal to account for potential preferences of exploration in the maze.

The experimenter running the RAWM assay was blinded to the genotype and surgery of the animals, and the data was collected through a video tracking and analysis setup (Ethovision XT 8.5, Noldus Information Technology, Leesburg, VA, USA). The program automatically analyzed the number of errors—an entry into an arm not containing the escape platform—per trial. The results of every three trials were averaged into a block to account for the large variability in performance; a training day thus consisted of 15 trials averaged into 5 blocks, and the memory probe consisted of 3 trials averaged into 1 block.

### 4.8. Statistical Analysis

All statistical analyses were performed with Graphpad Prism (Graphpad Software, La Jolla, CA, USA). Flow cytometry data for the time course of the peripherally-derived monocytes were analyzed by two-way analysis of variance (ANOVA), and significant main effects and interactions are reported. Two-way ANOVA was used for the blood monocyte flow cytometry with post hoc Bonferroni’s multiple comparisons. BrdU and EdU proliferation experiment data were analyzed by unpaired Student’s *t*-tests. RAWM data for blocks 1–10 were analyzed via two-way ANOVA, while block 11 memory probe data was analyzed by unpaired Student’s *t*-test. All data presented are means ± standard error of mean (SEM) with significance set at *p* < 0.05.

## Supplementary Materials

The following are available online at http://www.mdpi.com/1422-0067/19/6/1616/s1.
