# Supplementary material for: Persistent Infiltration and Impaired Response of Peripherally-Derived Monocytes after Traumatic Brain Injury in the Aged Brain
Source: Int J Mol Sci. 2018 May 30;19(6):1616. doi: 10.3390/ijms19061616 (PMC6032263; doi:10.3390/ijms19061616)
Supplement: Supplementary file 1 [file ijms-19-01616-s001.pdf]

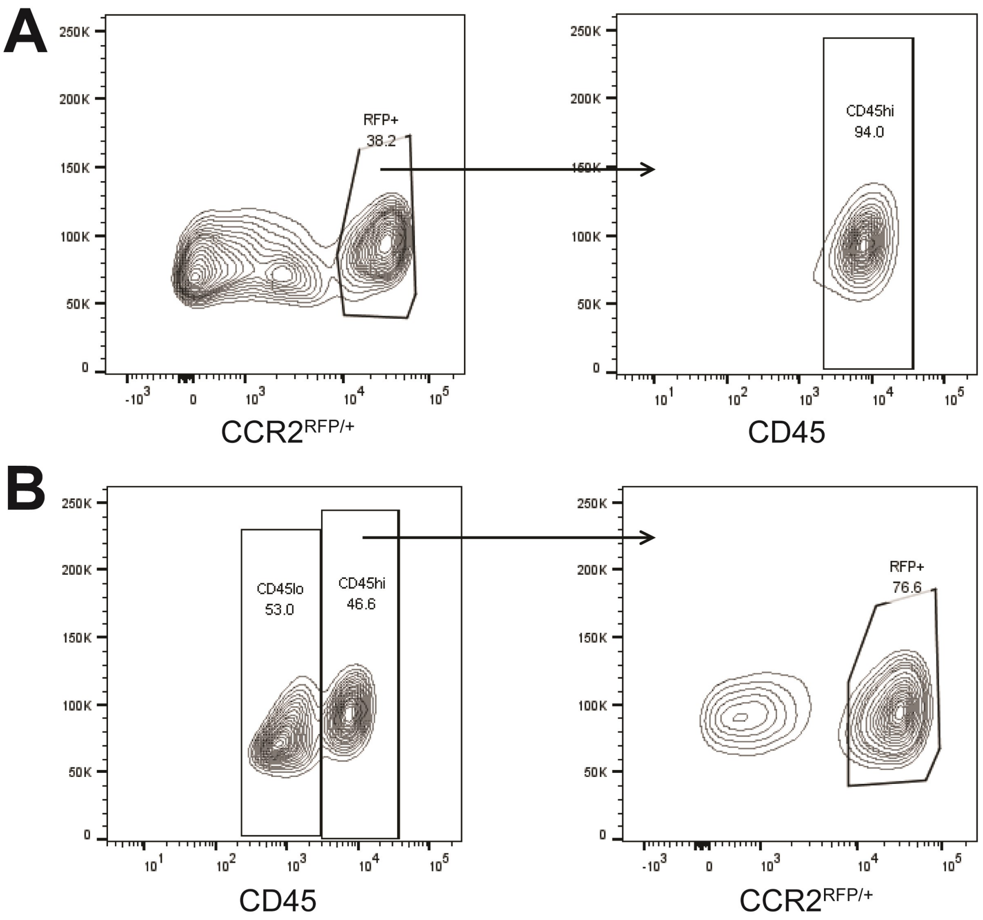

Figure S1. Majority of CCR2^{+} cells are CD45^{hi} and vice versa. **(A)** Representative flow cytometry profile showing that the majority of CCR2^{+} (gated from live, single, CD11b^{+} population from an injured, aged brain) monocytes are CD45^{hi}. **(B)** Representative flow cytometry profile showing that majority of CD45^{hi} monocytes gated from the same population as (A) are CCR2^{+}.

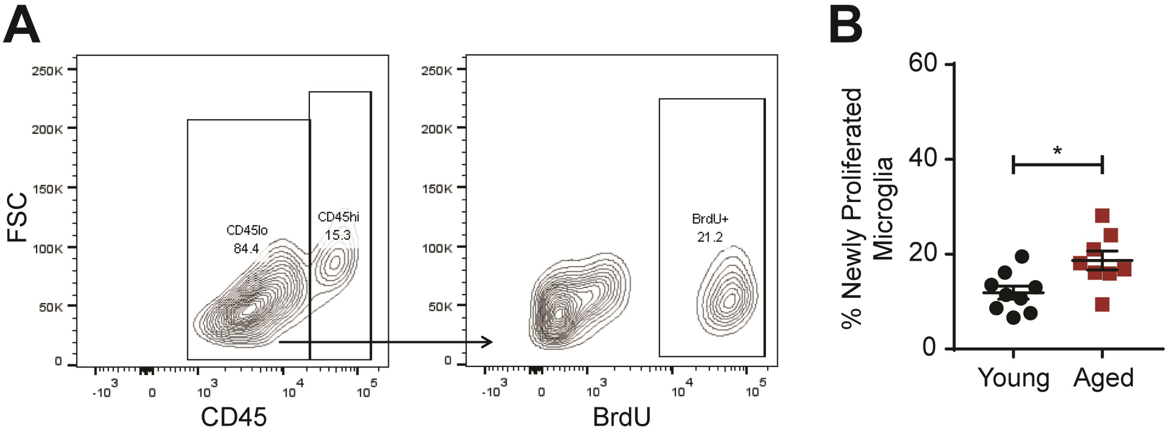

Figure S2. Age increases resident microglia (CD45^{lo}) proliferation in the injured brain at 4 dpi. **(A)** Representative flow cytometry profile for newly proliferated resident microglia. CD45^{lo} microglia were first gated from the CD11b^{+}, F4/80^{+} population (right). BrdU signal is then gated. Example images are from an aged animal. **(B)** Percent of resident microglia (CD45^{lo}) with BrdU^{+} signal. Age significantly increases the number of resident microglia that proliferated between 3 and 4 dpi. Data are means ± SEM (n = 8-9; Student's t-test, *$p<0.05$).

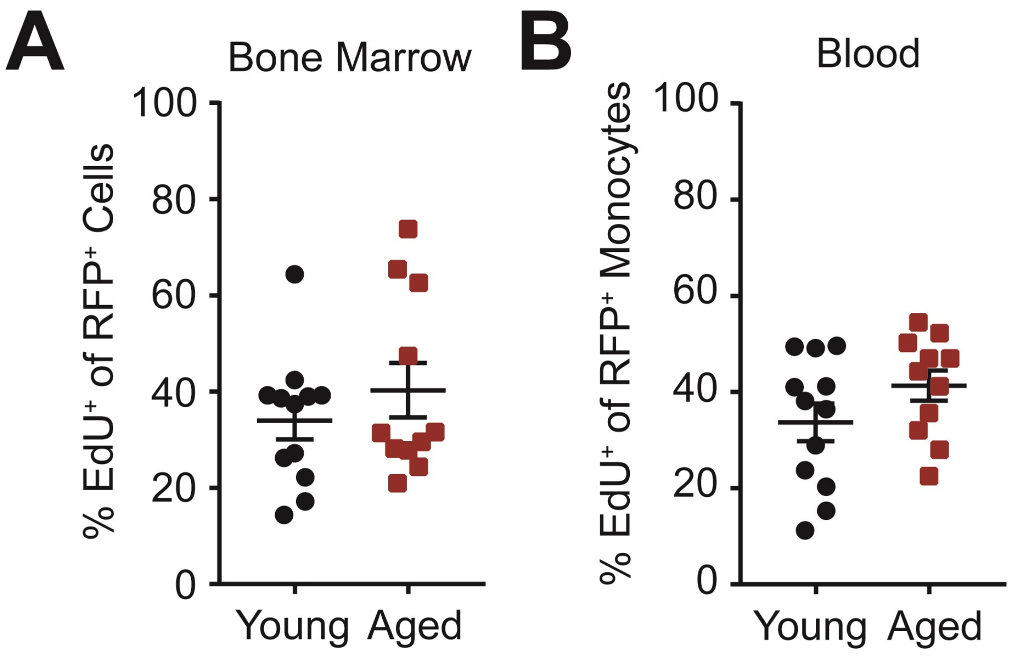

Figure S3. Age does not significantly increase new CCR2+ (RFP+) cells in the bone marrow or blood between 3 and 4 dpi. **(A)** Two injections of EdU were given to TBI animals between 3 and 4 dpi. EdU-positive cells were gated from CD11b+, CCR2+ populations from the bone marrow. Age did not significantly increase the proliferation of CCR2+ (RFP+) cells between 3-4 dpi (n=11-12). **(B)** CD11b+, CCR2+ cells were similarly gated from the blood. Age did not significantly increase the number of EdU-labeled monocytes in the blood (n=11-12; Student's t-test, p=0.14).

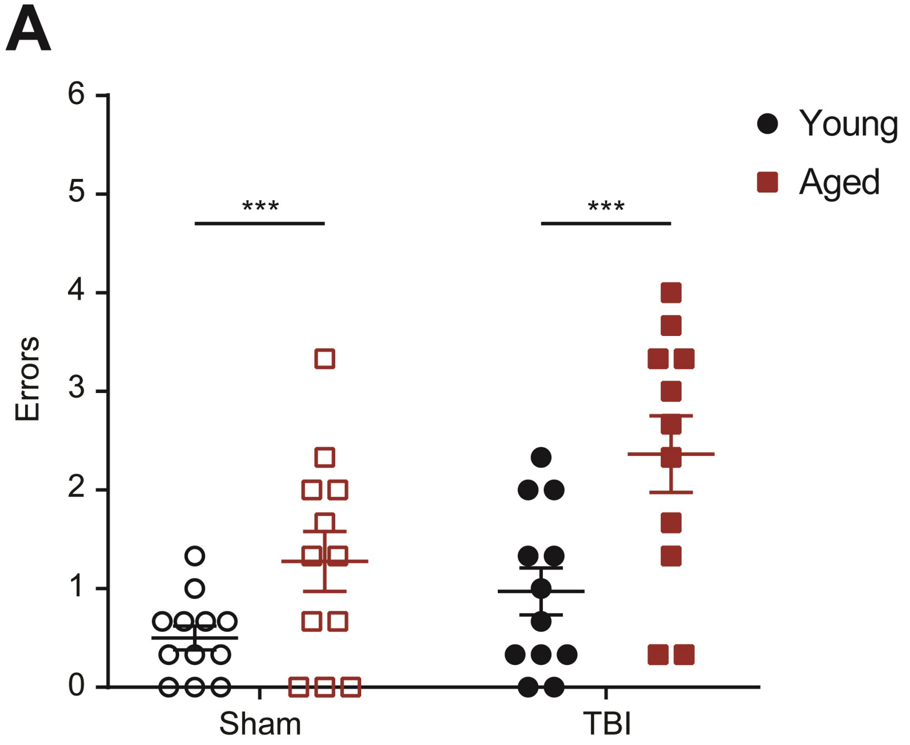

Figure S4. Age significantly impairs memory deficits on the radial arm water maze (RAWM) for sham and TBI animals at 30 dpi. **(A)** Individual animal performance during the memory test (block 11; 30 dpi). Injury and Age both increases the errors made. Aged, injured animals committed the most errors. (n=11-12; two-way ANOVA, significant main effects of Age and TBI, no significant Interaction, ***p<0.001).

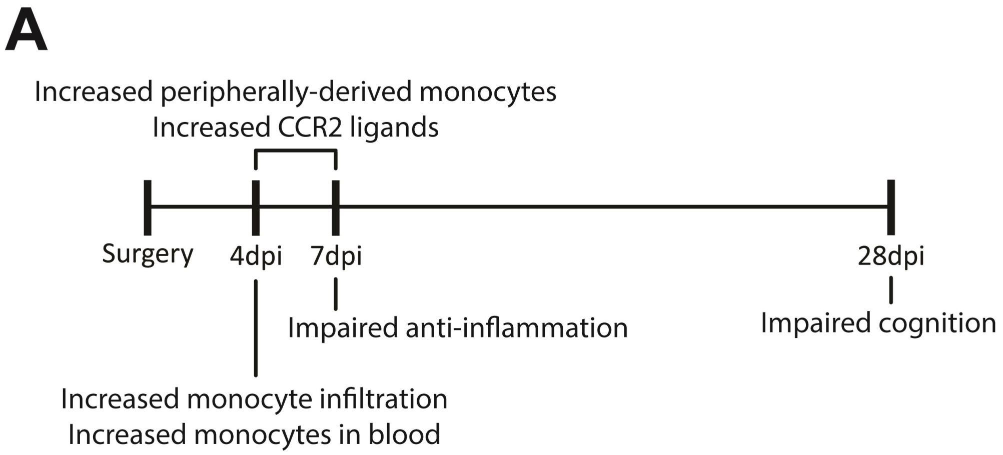

Figure S5. Time course of the present findings showing age-exacerbated TBI outcome.
